# Linking death registration and survey data: Procedures and cohort profile for The Irish Longitudinal Study on Ageing (TILDA)

**DOI:** 10.12688/hrbopenres.13083.2

**Published:** 2020-11-19

**Authors:** Mark Ward, Peter May, Robert Briggs, Triona McNicholas, Charles Normand, Rose Anne Kenny, Anne Nolan

**Affiliations:** 1 The Irish Longitudinal Study on Ageing, Trinity College Dublin, Dublin, Ireland; 2 Centre for Health Policy and Management, Trinity College Dublin, Dublin, Ireland; 3 Department of Medical Gerontology, St James's Hospital, Dublin, Ireland; 4 The Economic and Social Research Institute, Dublin, Ireland

**Keywords:** mortality, ageing, death certification, TILDA, data linkage

## Abstract

**Background:** Research on mortality at the population level has been severely restricted by an absence of linked death registration and survey data in Ireland. We describe the steps taken to link death registration information with survey data from a nationally representative prospective study of community-dwelling older adults. We also provide a profile of decedents among this cohort and compare mortality rates to population-level mortality data. Finally, we compare the utility of analysing underlying versus contributory causes of death.

**Methods:** Death records were obtained for 779 and linked to individual level survey data from The Irish Longitudinal Study on Ageing (TILDA).

**Results:** Overall, 9.1% of participants died during the nine-year follow-up period and the average age at death was 75.3 years. Neoplasms were identified as the underlying cause of death for 37.0%; 32.9% of deaths were attributable to diseases of the circulatory system; 14.4% due to diseases of the respiratory system; while the remaining 15.8% of deaths occurred due to all other causes. Mortality rates among younger TILDA participants closely aligned with those observed in the population but TILDA mortality rates were slightly lower in the older age groups. Contributory cause of death provides similar estimates as underlying cause when we examined the association between smoking and all-cause and cause-specific mortality.

**Conclusions:** This new data infrastructure provides many opportunities to contribute to our understanding of the social, behavioural, economic, and health antecedents to mortality and to inform public policies aimed at addressing inequalities in mortality and end-of-life care.

## Introduction

Linking data from death registers with survey and other individual-level data is commonplace in many countries. This practice has enabled a number of prospective cohort studies collecting rich individual-level data, such as the English Longitudinal Study on Ageing (ELSA) and the Health and Retirement Study (HRS), to examine associations between mortality and a wide range of factors (for example see: Lewer *et al.*, 2017; Wu *et al.*, 2016). The Republic of Ireland has lacked an equivalent data infrastructure and analyses of Irish mortality have therefore been largely limited to unlinked Census data ( Layte & Banks, 2016). Consequently, researchers’ ability to identify the determinants of mortality at the population level has been severely restricted.

In 2007, and again in 2017, the Central Statistics Office (CSO) conducted a limited data linkage exercise, linking all deaths that occurred in the year after the 2006 Census of Population to their Census record. However, these linked datasets are of limited utility due to the short one-year follow-up period and the very limited information collected as part of the census. Furthermore, both the census and mortality data files have limited socioeconomic status (SES) information, and no information on disease risk factors or antecedents ( CSO, 2010; CSO, 2019). Our linking of longitudinal survey and death register data enables us to supplement the rich data available from longitudinal surveys with detailed data on cause of death available from official mortality registers.

Previous research has highlighted the numerous limitations inherent in using unlinked Census data, including the long time between Census observation periods, and the dependence on unlinked numerators (count of deaths) and dominators (population grouping variable) ( Layte *et al.*, 2015; Layte & Banks, 2016; Layte & Nolan, 2016). Furthermore, Census data on SES variables in Ireland and elsewhere is particularly problematic due to the large amount of missing data. This missing data is often systematic being higher among younger age groups, women, and those not in paid employment at the time of Census data collection ( Layte & Nolan, 2016). Importantly, individuals with missing SES information have also been shown to have higher mortality rates, which means that previous research on the association between SES and mortality in Ireland will likely have underestimated the true strength of this association ( Layte & Banks, 2016). Beyond the issue of missing data inherent in analysing unlinked census data, even in cases were SES data is available, there is a large question mark over its validity. For example, White *et al.* ( White *et al.*, 2008) compared individual level social class from death records with that from the previous census in England and Wales and found that almost half of the records did not match. This incongruence is therefore another source of error. In light of the above, the necessity of linked survey-mortality data to properly identify the determinants of mortality rates is clear ( Mackenbach *et al.*, 2015).

As well as these problems with denominators, there may also be issues with the death counts themselves, particularly when interested in specific cause(s) of death rather than simply the event. For example, Daking and Dodds ( Daking & Dodds, 2007) found differences in ICD-10 coding between Australian Census and coroners’ data. Inconsistencies between population-based cancer registry data and death certificate data for cancer mortality have also been identified ( German *et al.*, 2011). A further complicating factor is that, in many cases, more than one condition may be compatible with the manner of death and indeed variability in the assignation of underlying cause of death has been well documented ( Danilova, 2016).

All of the above is not to say that death certificate records are themselves necessarily free of error. Indeed, coding and other errors have been widely documented ( Danilova *et al.*, 2016; Danilova, 2016; Harteloh, 2018; Harteloh *et al.*, 2010; McGivern *et al.*, 2017). These studies have highlighted numerous inconsistencies in both the recording of information on death certificates by physicians ( Myers & Farquhar, 1998) and coding practices across space and time ( Danilova, 2016), particularly at the most detailed level of ICD-10 codes. These inconsistencies are exacerbated when the goal is to identify an underlying cause of death when more than one condition is recorded on the death certificate ( Harteloh, 2018).

Here, we describe the steps taken to link death registration information with survey data derived from a large nationally representative prospective study of community-dwelling older adults. We also provide a profile of decedents among this cohort and compare mortality rates in this cohort to population-level data. Finally, we consider the utility of analysing underlying and contributory causes of death. This new data infrastructure provides many opportunities to contribute to our understanding of the social, behavioural, economic, and health antecedents to mortality and to inform public policies aimed at addressing inequalities in mortality and end-of-life care.

## Methods

### Death register data

Every death in the Republic of Ireland must be registered with the General Register Office (GRO). Registration is legally required, and non-registration is rare because of the necessity of a death certificate for many legal purposes. Firstly, the attending physician completes the medical certificate of the primary and contributory causes of death. This information, together with socioeconomic and demographic information provided by the next of kin or other qualified informant, is entered electronically at one of the 25 civil registration offices around the country and forwarded to the GRO. The GRO provides these records to the CSO on a weekly basis where it is collated for statistical reports on mortality. The CSO also administer a research micro-data file which includes individual-level data on date of death, residential address of decedent, place of death, primary and contributory causes of death, occupation of deceased, age of deceased, sex of deceased and marital status of deceased. All deaths registered on or after 1st January 2007 are coded according to ICD-10 rules. The CSO use Iris software to automatically assign ICD-10 to all diagnostic conditions and underlying cause of death from death certificates ( CSO, 2018).

### Survey data

The Irish Longitudinal Study on Ageing (TILDA) is a prospective nationally representative study of community dwelling adults aged ≥ 50 years resident in the Republic of Ireland. Details of the methodology employed by TILDA are fully described elsewhere ( Donoghue *et al.*, 2018; Kearney *et al.*, 2011; Kenny *et al.*, 2010; Whelan & Savva, 2013). Brieﬂy, TILDA participants were selected using multi-stage stratified random sampling whereby 640 geographical areas, stratified by socioeconomic characteristics, were selected, followed by 40 households within each area. The Irish GeoDirectory listing of all residential addresses provided the sampling frame. The first Wave of data collection was conducted between 2009 and 2011, with subsequent Waves collected at two-year intervals. Details of the sample maintenance strategies used by TILDA are also available elsewhere ( Donoghue *et al.*, 2017). TILDA collects information on a broad range of topics including health, economic, social, and family circumstances. Data collection consists of a number of components. Computer-assisted personal interviews (CAPI) and self-completion questionnaires (SCQ) were completed at each Wave of data collection and a comprehensive health assessment, conducted by trained nurses, was carried out at Waves 1 and 3, and will be repeated at Wave 6 in 2021. From Wave 2 onwards, End-of-Life (EOL) interviews have been completed with a spouse, relative, or friend in cases where a participant had passed away ( May *et al.*, 2017). TILDA is a member of the HRS family of studies and is therefore harmonised with a number of large prospective cohort studies on ageing including ELSA, HRS, and The Survey of Health, Ageing and Retirement in Europe (SHARE).

### Data linkage

Deaths among TILDA participants were identified by a number of methods. In many cases, spouses or other relatives of decedents contacted TILDA to inform them of the death of the participant. Other deaths were identified when interviewers visited the home of decedents to conduct subsequent waves of data collection. Also, where it was not possible to contact a participant, the TILDA data management team identified deaths through searches of the obituary website RIP.ie which is dedicated to publishing death notices in Ireland and deathevents.gov.ie, an online service that reports information on death events to public sector bodies. Finally, for a number of the remaining cases where the status of participants was not known, GRO records were interrogated in order to identify those who had died.

TILDA was granted approval from the GRO to link TILDA respondents to their corresponding death certificate information. As there is no unique personal identifier in Ireland that could be used to match TILDA decedents to their death certificate record, matching was performed on the basis of name, address and month/year of birth (and age, to account for possible misreporting of age and/or month/year of birth on either file). Where records could not be linked based on this information, additional information such as marital status was used. Data matching was conducted with the GRO in early 2018. Matching was performed for all individuals who died between Wave 1 (2009/2011) and March 2018. This procedure will be repeated as subsequent waves of TILDA data become available.

Matched death records were provided to TILDA in excel format. Each record consisted of a unique identifier, an immediate or proximal cause of death, and contributory factors. Of a total of 863 confirmed deaths among the TILDA sample, matching death records were obtained for 779 (90.3%) of all known deaths at that time. The 84 deaths not captured in this data linkage occurred after we completed the exercise and will be captured when we repeat data linkage in 2021. Table 1 shows the timing of all deaths among TILDA participants, including those for whom it was not possible to match to death records. The smaller number of deaths identified after Wave 4 is due to the fact that data linkage was carried out at the beginning of Wave 5 data collection.

**Table 1.** Timing of deaths in TILDA.

Timing of deaths	N	%
Deceased between Wave 1 & Wave 2	243	28.2
Deceased between Wave 2 & Wave 3	329	38.1
Deceased between Wave 3 & Wave 4	226	26.2
Deceased between Wave 4 & Wave 5	65	7.5
**Total**	863	100

### Coding of cause of death

Iris is a software tool for coding multiple causes of death and for the selection of the underlying cause of death. It is the preferred mortality coding tool of Eurostat. While early versions of Iris used the Centre of Disease Control-developed Medical Mortality Data System (MMDS), since version 5 it uses the Multicausal and Unicausal Selection Engine (MUSE). MUSE operates based on internationally agreed decision tables which are based on the most recent version of ICD-10. We used Iris version 5.4.0. The Iris software is free to use and can be downloaded, along with supporting materials, from the Iris institute.

Firstly, Iris attempts to code all diagnostic expressions included in each death certificate according to the World Health Organisation (WHO) ICD-10 classification system. Once all diagnostic expressions have been assigned an ICD-10 code, Iris then selects an underlying cause according to the MUSE decision tables which are regularly reviewed by the Iris consortium. Iris also provides a text format explanation on how the WHO mortality coding guidelines were applied when assigning underlying cause from the list of diagnostic conditions. Where possible, each condition reported in the death records were coded at the four-digit ICD-10 level. In cases where this automated coding system fails to assign an ICD-10 code or an underlying cause, manual coding was required. In our case, Iris successfully coded 18% of the 1,605 diagnostic expressions in the first iteration and assigned an underlying cause to 5.3% of the cases.

### Underlying cause of death

We have operationalised underlying cause of death according to the WHO definition as “ *the disease or injury which initiated the train of morbid events leading directly to death, or the circumstances of the accident or violence which produced the fatal injury”* ( United Nations, 1991). We grouped underlying causes of death to ICD-10 chapters in order to adhere to TILDA data protection policies regarding minimum cell sizes for reporting purposes and also to ensure that groupings were large enough to enable statistically robust analyses. Of the 779 deaths, cancer was identified as the underlying cause of death for 37.0%; 32.9% of deaths were attributable to diseases of the circulatory system; 14.4% due to diseases of the respiratory system; while the remaining 15.8% of deaths occurred due to all other causes ( Table 2).

**Table 2.** Distribution of main ICD-1O Chapters classification among TILDA decedents.

ICD-10 chapters	ICD-10 code	% (n)
Neoplasms	C00-D49	37.0 (288)
Diseases of the circulatory system	I00-I99	32.9 (256)
Diseases of the respiratory system	J00-J99	14.4 (112)
All others		15.8 (123)
Total		100 (779)

## Statistical analysis

Descriptive statistics included counts, percentages, and 95% confidence intervals. We used Cox proportional hazards regression models to estimate sex-adjusted hazard ratios for smoking as a risk factor for cause-specific mortality. Respondents lost to follow up were right-censored at the end of the follow-up-period (March 2018). The results of this analysis are presented in Figure 3. All analyses were conducted using Stata/MP 14.2 ( StataCorp, 2015).

## Results

### Description of sample

For reference, the distribution of important socio-demographic characteristics of the full TILDA sample and those who died over the course of the study are presented in Table 3. The mean age of TILDA participants at baseline was 64 years (95% CI: 63.6, 64.3); 51.8% were women (95% CI: 50.7, 52.8). Almost one-third (31.5%, 95% CI: 30.0, 33.1) had primary level education while 22.2% had completed tertiary education (95% CI: 21.0, 23.5). A similar proportion of participants were employed (36.0%, 95% CI: 34.5, 37.4) or retired (36.6%, 95% CI: 35.1, 38.1) with the remainder unemployed, in full-time education or training, permanently sick or disabled, or looking after the family home on a full-time basis. In terms of household social class, 25.1% (95% CI: 23.8, 26.5) of participants were in the professional, managerial or technical social class while 21.0% (95% CI: 19.7, 22.4) in the semi- or un-skilled class. The remaining unclassified group included participants for whom there was not enough information to assign to a social class and those who were never economically active. The mean annual household income was €34,285.

**Table 3.** Distribution of key sample characteristics for baseline sample, all-cause and cause-specific mortality.

	Baseline sample (n=8,174) % (95% CI)	All-cause mortality (n=779) % (95% CI)	Cancers (n=288) % (95% CI)	Circulatory (n=256) % (95% CI)	Respiratory (n=112) % (95% CI)	Other causes (n=123) % (95% CI)
**Mean age**	64.0 (63.6,64.3)	75.3 (74.3,76.3)	72.2 (70.8,73.7)	77.4 (75.8,79.0)	77.8 (75.3,80.3)	74.6 (72.1,77.1)
**Men**	48.2 (47.2,49.3)	53.1 (48.4,57.8)	53.7 (46.3,61.0)	56.5 (48.7,63.9)	40.9 (29.8,52.9)	55.4 (43.7,66.6)
**Women**	51.8 (50.7,52.8)	46.9 (42.2,51.6)	46.3 (39.0,53.7)	43.5 (36.1,51.3)	59.1 (47.1,70.2)	44.6 (33.4,56.3)
**Education**						
**Primary**	31.5 (30.0,33.1)	53.1 (48.6,57.6)	45.2 (38.1,52.4)	59.9 (52.1,67.1)	56.0 (43.9,67.5)	52.3 (41.3,63.1)
**Secondary**	46.3 (44.9,47.7)	34.8 (30.6,39.3)	40.1 (33.3,47.3)	31.4 (24.7,38.9)	33.5 22.7,46.4)	32.4 (23.2,43.2)
**3 ^rd^ level**	22.2 (21.0,23.5)	12.1 (9.7,14.9)	14.8 (11.1,19.4)	8.8 (5.7,13.4)	10.5 (5.1,20.2)	15.3 (9.4,23.9)
**Principal** **economic** **status**						
**Employed**	36.0 (34.5,37.4)	11.4 (9.0,14.3)	14.4 (10.0,20.3)	10.5 (6.9,15.6)	8.1 8.1 (3.5,17.4)	9.8 (5.2,17.7)
**Retired**	36.6 (35.1,38.1)	64.6 (60.3,68.7)	64.1 (56.9,70.6)	64.6 (57.3,71.4)	68.7 (56.0,79.1)	61.8 (49.8,72.5)
**Other** *	27.5 (26.2,28.8)	24.0 (20.4,28.0)	21.5 (16.3,27.8)	24.8 (18.7,32.1)	23.2 (14.1,35.8)	28.4 (19.1,40.0)
**Occupation** **social class**						
**Professionals**	25.1 (23.8,26.5)	16.9 (13.4,21.0)	25.0 (18.3,33.3)	12.2 (7.9,18.5)	9.9 (3.9,23.1)	18.0 (10.1,30.0)
**Non-manual**	28.7 (27.4,29.9)	29.0 (24.3,34.2)	27.6 (20.2,36.3)	33.0 (25.1,42.1)	23.9 (13.7,38.2)	26.8 (16.2,40.9)
**Skilled manual**	17.5 (16.4,18.7)	17.1 (13.3,21.8)	21.7 (14.9,30.4)	14.8 (9.5,22.4)	12.1 (5.8,23.4)	18.0 (9.9,30.6)
**Semi- & un-** **skilled**	21.0 (19.7,22.4)	21.8 (17.7,26.7)	16.0 (10.0,24.5)	24.7 (17.5,33.7)	29.0 (17.8,43.4)	20.2 (11.3,33.4)
**Not classified**	7.7 (6.9,8.6)	15.1 (11.4,19.9)	9.7 (5.5,16.5)	15.2 (9.5,23.6)	25.1 (13.7,41.5)	17.0 (8.3,31.6)
**Mean** **household** **income**	€34,285 (32526,36043)	€21,184 (18762,23606)	€23,547 (17595,29499)	€19,024 (16154,21894)	€20,344 (17055,23632)	€22,074 (18337,25812)

* The Other occupational group includes: unemployed, in full-time education or training, permanently sick or disabled, or looking after family home on a full-time basis.

Overall 9.1% of TILDA participants died during the nine-year follow-up period and the average age at death was 75.3 years (95% CI: 74.3, 76.3). The average age at death from cancers was 72.2 years (95% CI: 70.8, 73.7); diseases of the circulatory system 77.4 years (95% CI: 75.8, 79.0); and diseases of the respiratory system 77.8 years (95% CI: 75.3, 83.0). Mortality rates were higher among less educated participants, manual occupation social class groups, and those with lower average annual household incomes.

### Comparison of mortality rates to CSO life tables

**Figure 1.**
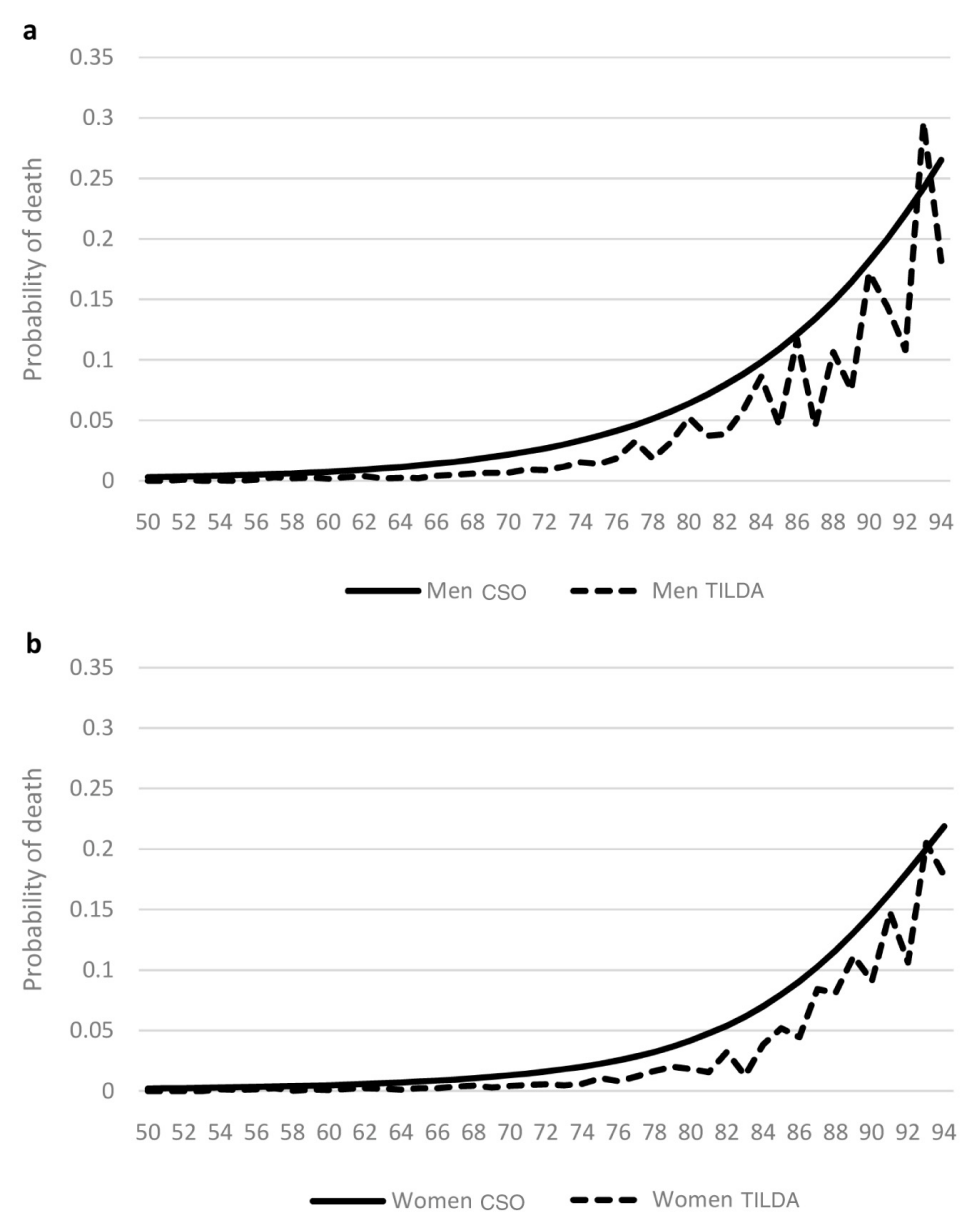
TILDA mortality by age compared to CSO life tables 2010–2012. ( **a**) Male mortality; ( **b**) female mortality.

In order to assess the representativeness of the TILDA mortality data in the Irish population, we compared our data to the Census of Population life tables. For this exercise, we used un-weighted data so that every death was counted equally. Figure 1a, b show the mortality rate for men and women, respectively, with CSO life tables for 2010–2012. The mortality rate on the y-axis was based on the hazard function which was calculated as the number of deaths at age x / the number of persons surviving to exact age x out of the original 100,000 aged 0. The x-axis was truncated at 94 years due to the small number of deaths that occurred after that age. Overall, mortality rates among younger TILDA participants aligned more closely than those among older decedents, with those observed in the population. This pattern is similar to that reported by the Health and Retirement Study (Weir, 2016).

**Figure 2.**
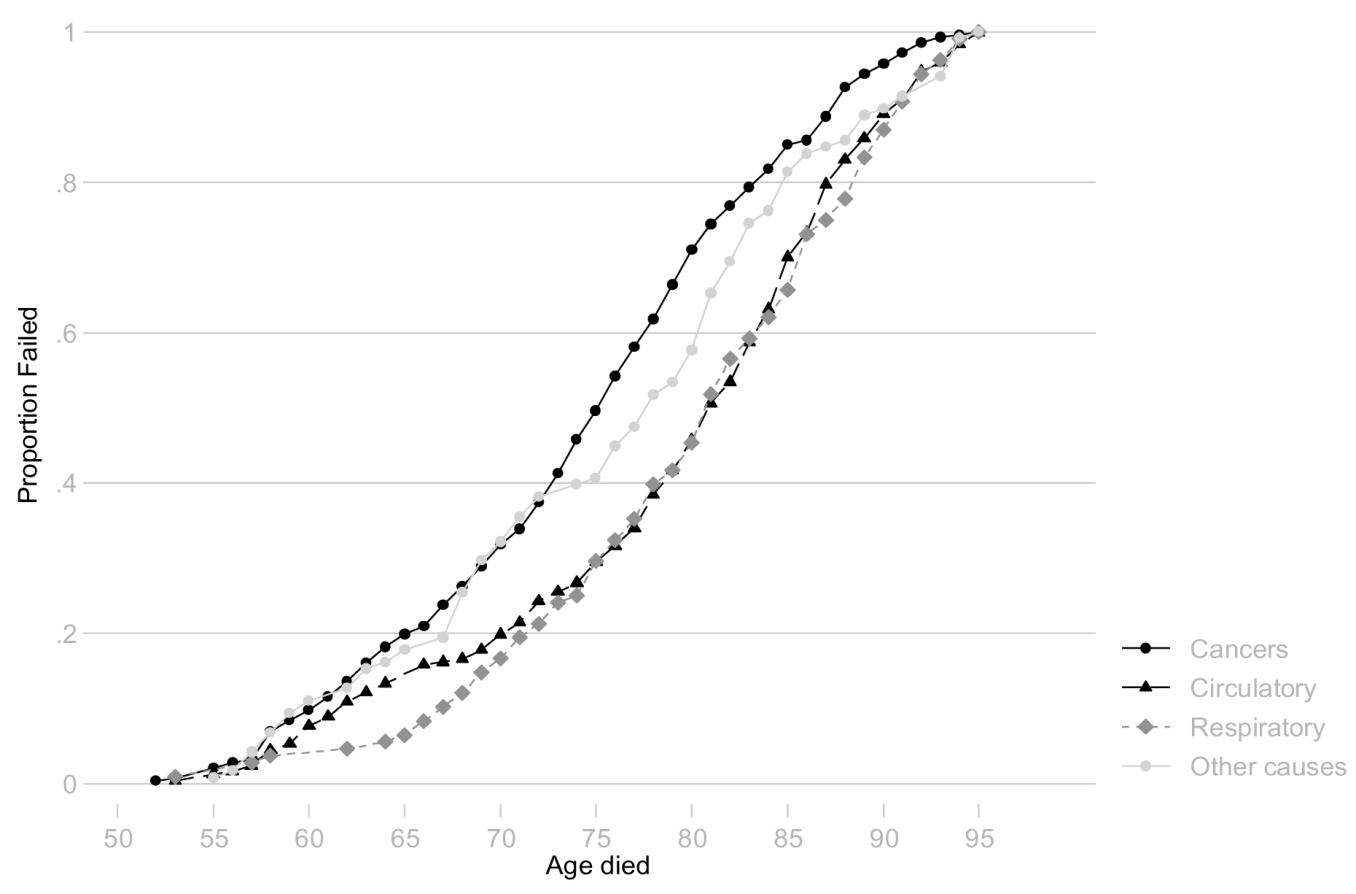
Cause-specific failure curves.

Figure 2 shows the cause-specific failure curves for the major disease groups which highlight important differences. There were fewer deaths due to diseases of the respiratory system, particularly before 70 years of age. Most of the deaths before this age occurred due to neoplasms and other causes including accidental deaths. After 70 years, a similar pattern was observed for diseases of the circulatory and respiratory system while neoplasms accounted for the greatest number of deaths.

**Figure 3.**
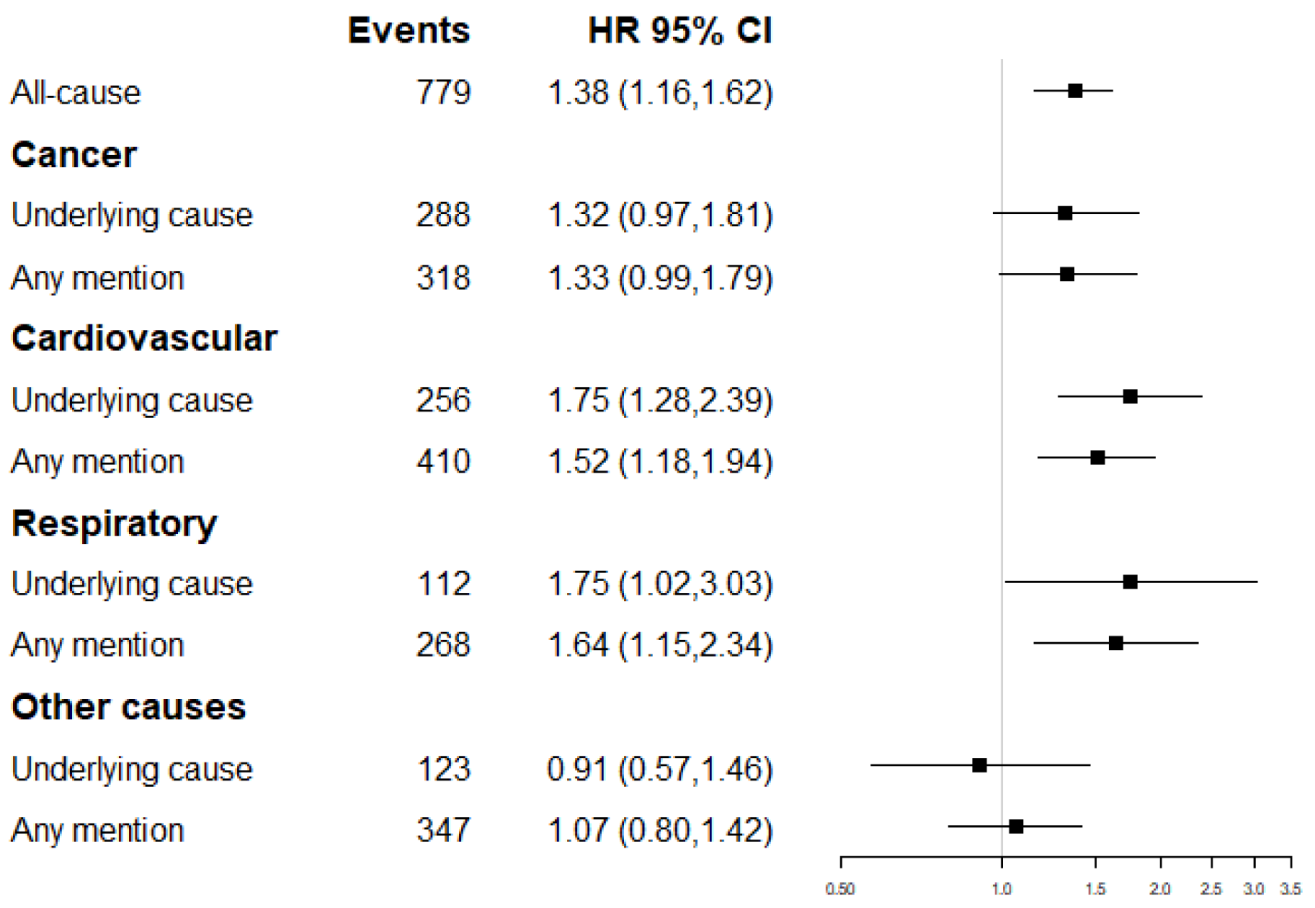
Sex-adjusted hazard ratios for ever smoking in relation to underlying and contributory cause-specific mortality.

### Underlying versus contributory cause of death

As well as the underlying cause of death described above, the death certificates also contained information on other diseases, injuries, or events that contributed to death. A contributory cause of death is a disease or condition that contributed to the death but was not directly implicated and recorded in part two of death certificates. While this information has been rarely used in epidemiological research, recent evidence suggests that it may have some methodological utility ( Batty *et al.,* 2019). For present purposes, contributory causes include diseases and conditions listed anywhere on the death certificate.

Among the 779 death records, up to seven contributory causes were also recorded and 67.5% of records had at least one contributory cause listed. One of the key advantages of our approach to data linkage is that we were able to assign an ICD-10 code to every contributory cause of death, thus enabling us to consider these contributory factors as well as the underlying cause of death. Through this procedure we identified neoplasms as being a contributory factor in 40.8% of deaths, while diseases of the circulatory system and diseases of the respiratory system were mentioned in 52.6% and 34.4% respectively ( Table 4).

**Table 4.** Distribution of main ICD-1O Chapters classification of underlying cause of death and contributory diseases among TILDA decedents.

ICD-10 chapters	Underlying cause % (n)	Contributory % (n)
Neoplasms	37.0 (288)	40.8 (318)
Diseases of the circulatory system	32.9 (256)	52.6 (410)
Diseases of the respiratory system	14.4 (112)	34.4 (268)
All others	15.8 (123)	44.5 (347)
Total	100 (779)	

To assess the utility of contributory cause of death versus underlying cause, Figure 3 shows the sex-adjusted hazard ratios for smoking as a risk factor for all-cause, and cause-specific mortality according to both underlying and contributory (any mention) cause of death. We chose smoking to test our hypothesis that similar estimates would be derived from both underlying and contributory conditions as smoking is an established risk factor for mortality and it has been used for a similar purpose previously ( Batty *et al.*, 2019). In each instance, we observed similar estimates whether we assigned death due to an underlying or contributory cause. Smokers, including those who had quit, had an increased all-cause mortality risk (HR= 1.38, 95% CI:1.16-1.62) compared to participants who never smoked. The estimates for both cardiovascular and respiratory, contributory (any mention) and underlying cause of death were similar. The precision of the estimates was better when including the contributory conditions due to the increased number of cases included in these groups.

## Discussion

We have described the procedures employed to link death registration information to survey data among a large sample derived from a nationally representative cohort of community-dwelling older adults. From the first round of data collection in TILDA to early 2018 (nine-year follow-up), it was possible to link to death registration data of 779 confirmed deaths. This compares favourably to a similar exercise conducted by the CSO whereby all deaths occurring in the year after the 2006 Census of Population were matched to their corresponding Census record which resulted in a matching rate of 79.8%. The Northern Ireland Mortality Study, which links death certificate information with the 1991, 2001 and 2011 UK Census of Population, obtained a matching rate of 94% using names and addresses ( CSO, 2010).

Comparison with life tables from the CSO showed that mortality rates among younger participants closely aligned with those in the wider population. While TILDA mortality rates were lower in the older age groups, this divergence is unsurprising given that the TILDA sample was drawn from adults living in the community which means that they were on average healthier than the total population of older adults. Furthermore, this pattern is similar to that reported from the Health and Retirement Study ( Weir, 2016).

There are a number of important advantages to the approach to data coding and linkage described here. Having access to detailed death registration information provides us the opportunity to operationalise the causes of mortality in a number of different ways: underlying all-cause and cause-specific, contributory, and multiple cause of death. The richness and breadth of information collected by TILDA over multiple waves provides us with a unique opportunity to contribute to the study of mortality.

Having complete death registration data is particularly important when concerned with assessing multiple causes of death. For example, recent studies demonstrated how a multiple-cause-of-death approach is useful to characterise the contribution of diabetes ( Rodriguez *et al.*, 2019) and falls ( Kiadaliri *et al.*, 2019) to mortality. Here, we assessed the utility of contributory cause of death versus underlying cause of death using the example of smoking as a risk factor for cause-specific mortality. We observed similar estimates whether we assigned death due to an underlying or contributory cause, which suggests the use of either contributory or underlying cause may not greatly impact on estimates of the association between risk factors and mortality. This finding is similar to that reported previously by Batty *et al.* (2019) and an earlier study by Crews *et al.* (1991). Indeed, one potential benefit of using contributory causes is increased statistical power due to larger numbers and a reduction in the associated error. More broadly, the utility of contributory cause of death in epidemiological research has also been shown to be similar to that of underlying cause while reducing the risk of measurement error due to the potential identification of an underlying cause.

The application of standardised coding dictionaries and decision tables in the Iris software can aid harmonisation across data sources and jurisdictions. This harmonisation is critical to enable researchers better understand differences in the mortality rates and the mechanisms that explain differences between populations. However, our initial application of IRIS software for assigning ICD-10 codes to all conditions contained in the death registration data and subsequently identifying an underlying cause of death required substantial manual input. The failure to automatically assign codes was due mostly to syntax and semantic differences between the terms included on death certificates and the Iris dictionary. For example, Iris failed to automatically code cases of “ischaemic heart disease” as it searched for “ischemic”. When such failures occurred, researchers had to manually enter the appropriate ICD-10 code. The Iris dictionary was then amended so that subsequent incidences of ischaemic heart disease were automatically coded. This procedure will greatly improve the automation of the coding process in future waves of TILDA.

### Limitations

While every effort has been made to ensure that an appropriate underlying cause of death was assigned to each decedent, we cannot account for potential errors in the recording of individual death certificates. For example, a comparison of death certificate data with associated medical records showed high error rates on death certificates, including ICD-10 coding ( McGivern *et al.*, 2017). However, our application of broader diagnostic categories in the form of ICD-10 Chapters and our ability to include contributory conditions and multiple-cause-of-death in our analyses should minimise the impact of these potential errors. For example, consistency in coding of mortality has been shown to improve when cause of death is grouped into broad diagnostic categories ( Danilova, 2016).

There is necessarily a time lag whereby, unbeknownst to us, participants may have died since the last round of data collection. This is inevitable as we do not have an automated linkage system with the GRO. The practical effect of this is that we have likely underestimated the rates of mortality for the most recent period. The potential impact of this on our current analyses will be assessed during subsequent rounds of data linkage.

## Conclusion

This is the first time that death registration data has been linked to survey data in the Republic of Ireland. This work therefore provides an important data infrastructure for research on mortality in Ireland. The rich and wide-ranging data collected by TILDA, including objective health assessment data, means that we have a unique opportunity to contribute to our understanding of the social, behavioural, economic, and health antecedents to mortality and to inform public policies aimed at addressing inequalities in mortality and end-of-life care. Finally, because TILDA is harmonised with other large prospective cohort studies within the HRS family of studies, this new data infrastructure also provides opportunities for researchers and policy makers interested in examining difference in the nature of mortality and its antecedents between populations.

## Data availability

### Underlying data

The first four waves of TILDA data are available from the Irish Social Science Data Archive (ISSDA) at www.ucd.ie/issda/data/tilda/. Due to the sensitive nature of death registration data, the cause of death data reported here are not publicly accessible at this time. Requests to access this data can be made directly to TILDA (tilda@tcd.ie) and will be considered on a case-by-case basis.

To access the TLDA survey data, please complete an ISSDA Data Request Form for Research Purposes, sign it, and send it to ISSDA by email ( issda@ucd.ie).

For teaching purposes, please complete the ISSDA Data Request Form for Teaching Purposes, and follow the procedures, as above. Teaching requests are approved on a once-off module/workshop basis. Subsequent occurrences of the module/workshop require a new teaching request form.
