# Supplementary material for: Linking death registration and survey data: Procedures and cohort profile for The Irish Longitudinal Study on Ageing (TILDA)
Source: HRB Open Res. 2020 Nov 19;3:43. Originally published 2020 Jul 8. [Version 2] doi: 10.12688/hrbopenres.13083.2 (PMC7376615; doi:10.12688/hrbopenres.13083.2)
Supplement: Peer review history

# Peer review: Reviewer response for version 2

**DOI:** 10.21956/hrbopenres.14315.r28409

**Published:** 2020-11-23

**Authors:** Zubair Kabir

**Affiliations:** 1 School of Public Health, University College Cork, Cork, Ireland

The authors have adequately addressed all my previous concerns. Happy to approve this version.

 Well done!

Is the rationale for developing the new method (or application) clearly explained?

Yes

Is the description of the method technically sound?

Yes

Are the conclusions about the method and its performance adequately supported by the findings presented in the article?

Yes

If any results are presented, are all the source data underlying the results available to ensure full reproducibility?

Partly

Are sufficient details provided to allow replication of the method development and its use by others?

Partly

Reviewer Expertise:

Tobacco Control; Non-communicable epidemiology; Global Burden of Disease (GBD) methodology.

I confirm that I have read this submission and believe that I have an appropriate level of expertise to confirm that it is of an acceptable scientific standard.

# Peer review: Reviewer response for version 2

**DOI:** 10.21956/hrbopenres.14315.r28410

**Published:** 2020-11-19

**Authors:** Dan Lewer

**Affiliations:** 1 Department of Epidemiology and Public Health, University College London, London, UK

No further comments.

Is the rationale for developing the new method (or application) clearly explained?

Yes

Is the description of the method technically sound?

Yes

Are the conclusions about the method and its performance adequately supported by the findings presented in the article?

Yes

If any results are presented, are all the source data underlying the results available to ensure full reproducibility?

Yes

Are sufficient details provided to allow replication of the method development and its use by others?

Yes

Reviewer Expertise:

Research using electronic health records; public health; health and social exclusion; health inequalities.

I confirm that I have read this submission and believe that I have an appropriate level of expertise to confirm that it is of an acceptable scientific standard.

# Peer review: Reviewer response for version 1

**DOI:** 10.21956/hrbopenres.14183.r27633

**Published:** 2020-07-31

**Authors:** Zubair Kabir

**Affiliations:** 1 School of Public Health, University College Cork, Cork, Ireland

This is an important piece of linkage study that is relevant to the Irish context when such data linkages are available elsewhere. It is also important to note that linkage studies are methodologically challenging in Ireland because of the lack of a unique identifier. The CSO did make attempts earlier to undertake such linkage research but was insufficient and was both labour and resource intensive. The current study builds on earlier linkage studies undertaken both by CSO and GRO in 2013 and 2018, respectively.

 My main concern is the lack of explicit description of the linkage methodology in the current paper, which will not be very helpful for a researcher towards reproducibility. There are currently no standardized quality appraisal tools available to assess quality and bias of any linkage studies. However, it is essential that a linkage study must meet the following characteristics:

- Completeness of source databases
- Accuracy of data sources
- Linkage methodology and technology
- Ethical and data security considerations.

In the context of the current study - the first two criteria are broadly met. However, my main concern is with the linkage methodology and technology. My understanding is that the TILDA researchers were not primarily involved in the linkage methodology given that matching of records were undertaken separately by CSO in 2013 and by GRO in 2018. The TILDA team had a role to get an approval and forward their data to these two data sources team who in fact undertook the matching process - the details of which are not available to us. It also appears that the technology (software) used is IRIS, which is a broadly validated accepted tool for coding purposes employed by EUROSTAT and CSO in the past. However, this software also had limitations in capturing and coding all the diagnostic expressions - only 18% and 5% of all the cases. The rest of the matching was done manually - by whom and how is unclear. This is a crucial step for which sufficient information and clarity is lacking. Second, the matching was not 100% accurate - around 10% of records were unmatched - and further analyses of these unmatched records are essential to rule out systematic bias - measurement error, and such sensitivity analyses (false positives and false negatives) have not been provided. Third, the matching variables employed were only three - name, address, and age (and marital status for some, but not sure for how many?). Names, especially for females can change once married; addresses are not always permanent - and age is also variable. Therefore, further details on how these methodological limitations during the process of matching were handled are unclear. There is also limited information on ethical and data security considerations for this linkage study when personal data have been used, especially from a GDPR perspective.

 Furthermore, the coding practices of causes of death are crucial for any linkage studies. The authors have undertaken a separate analysis of exploring contributory versus underlying causes of deaths for the participants, and I believe that this piece of research is the sole contribution of the TILDA team to this paper. However, this could have been explained further and there is lack of clarity on how the unclassified causes of deaths within each of the three main types of causes of deaths (cancer, cardiovascular and respiratory) were handled. The CSO website clearly indicates ‘unclassified’ causes of cancer deaths and likewise for other conditions - and the Global Burden of Disease (GBD) Study team call these as ‘garbage’ codes. The GBD studies on causes of death have shown that there is a good proportion of ‘garbage’ codes for any death registry, and they have also developed a statistical technique on how to ‘redistribute’ these garbage codes. No such information is available to us in the current study.

 In short, I approve the study but has methodological limitations and caveats which could have been addressed.

Is the rationale for developing the new method (or application) clearly explained?

Yes

Is the description of the method technically sound?

Yes

Are the conclusions about the method and its performance adequately supported by the findings presented in the article?

Yes

If any results are presented, are all the source data underlying the results available to ensure full reproducibility?

Partly

Are sufficient details provided to allow replication of the method development and its use by others?

Partly

Reviewer Expertise:

Tobacco Control; Non-communicable epidemiology; Global Burden of Disease (GBD) methodology.

I confirm that I have read this submission and believe that I have an appropriate level of expertise to confirm that it is of an acceptable scientific standard, however I have significant reservations, as outlined above.

# Response

**Published:** 2020-11-06

**Authors:** Mark Ward

**Affiliations:** The Irish Longitudinal Study on Ageing, Trinity College Dublin, Ireland

**Response to Reviewer 1 comments – Dan Lewer**

**Comment 1.** Thank you for inviting me to review this article. It provides a clear summary of a linkage exercise conducted between a community health survey of older people and national mortality data in Ireland. The data is a valuable resource and researchers will find this technical article useful.

 To my knowledge this type of data is not commonplace (as per first line of introduction), which strengthens the international importance of this data.

**Response 1.** Thank you for taking the time to review our manuscript and providing insightful comments. Indeed, this is the first time that this data linkage exercise has been conducted in the Republic of Ireland and as such we hope that it will be a valuable resource for researchers who wish to better understand the antecedents of mortality among older adults.

**Comment 2.** I think a central use of this data is analyses of the association between longitudinal information on exposures and mortality (e.g. what is the effect of weight loss, quitting smoking, or cognitive decline?).

 This is not discussed in the article, and I think it might be worth mentioning this as a potential use of the dataset. In general, I would find it useful to know some of the key research questions that the authors think the dataset might address (though of course it's not possible to anticipate all the different research uses).

**Response 2. **This data linkage exercise was the first step in a wider programme of research being conducted within TILDA. This research is funded by the Health Research Board (ILP-PHR-2017-022)

 The project is titled “Do we die as we live? Age, socioeconomic status, healthcare utilisation and pathways to death in Ireland” and is led by Professor Rose Anne Kenny (PI, TCD) and Dr Anne Nolan (Lead applicant, ESRI).

 Three broad research questions are being examined in this project:

 1) How do patterns of all-cause, cause-specific and amenable mortality in the over 50s in Ireland vary across groups defined by socioeconomic status, co-existing conditions, and cause of death?

 2) What are the possible mechanisms (e.g., underlying health conditions, differential health behaviours, accessibility of healthcare services, etc.) that underlie these patterns?

 3) What are the determinants of healthcare utilisation and costs at the end of life among the over 50s in Ireland?

**Comment 3. **What is a confirmed death? If not from the linked mortality records, how do you find out that a participant has died (i.e. how do you know that 863 participants died?). Apologies if I missed an explanation of this in the text.

**Response 3. **Deaths among TILDA participants were identified through a number of sources. In many cases, spouses or other relatives of decedents contacted TILDA to inform the research team of the death. Other deaths were identified when interviewers visited the home of decedents to conduct subsequent waves of data collection. Also, where it was not possible to contact a participant, the TILDA data management team identified some deaths through searches of the obituary website dedicated to publishing death notices in Ireland, RIP.ie. Finally, in the remaining cases where the status of participants were not known, GRO records were interrogated in order to identify those who had died.  We have now included text to reflect this in the ‘data linkage’ section on page 4.

**Comment 4.** Is it worth adding some information on the associations with successful linkage? (i.e. were certain types of participant less likely to be linked?).

**Response 4.** On reflection our referring to the 863 total deaths among TILDA participants has led to some confusion. The 779 death records that we successfully matched were all the deaths confirmed by us at the time we carried out data linkage. The remaining 84 (863 - 779) deaths occurred after we had requested the death records from the GRO. These included the 65 deaths noted in Table 1 that occurred between waves 4 and 5 of TILDA data collection. We fully expect that the death records of these individuals will be included in the next round of data linkage in 2021.

 We have now included the following text where we describe Table 1: *“The 84 deaths not captured in this data linkage occurred after we completed the exercise and will be captured when we repeat data linkage in 2021”.*

**Comment 5.** For participants who are linked, what is the probability of correct linkage? Did the linkage process use an existing method, and is there any validation that the linkages are correct?

**Response 5.** Unfortunately we have no way of checking this. However, we are confident that the participants we have linked were correct. As described in the text we used a number of participant characteristics to ensure that we correctly identified individuals – “name, address and month/year of birth (and age, to account for possible misreporting of age and/or month/year of birth on either file). Where records could not be linked based on this information, additional information such as marital status was used.” Furthermore, as discussed in response to comment 3, in many cases this information was confirmed by a family member prior to the linkage exercise. Of course, every care was taken to ensure the accuracy of the characteristics used to identify death records in the GRO files.

 As also noted in the manuscript, Ireland does not have a unique health identifier which could have been used for the purpose of matching participant records, nor is there an automated notification of death available to use. The latter is the method used by a number of similar cohort studies to identify deaths among their participants.

**Comment 6.** I like the analysis of smoking. It might be worth adding a brief justification for this analysis to the introduction (e.g. that the relationship between smoking and different causes of death is well-researched in other sources, so it acts as a kind of validation - you would expect a stronger association between smoking and respiratory causes of death than between smoking and all-cause mortality; or because it allows you to evaluate the difference between the derived 'underlying cause' of deaths and contributing causes?). Would it be possible to add the association between ever-smoking and all-cause mortality to figure 3 for comparison?

**Response 6. **This is an excellent suggestion. Thank you.

 This particular analysis was informed by similar work carried out using UK Biobank data by Batty et al. The aim of this research, and our aim also, was to assess the utility of cause of death data extracted from the underlying cause field versus any location on the death certificate. The estimates do also confirm a stronger association between smoking and respiratory causes of death compared to all-cause mortality which is re-assuring but was not our main aim in this analysis.

 Our choice of smoking as a risk factor was, as you identify, because it is so well established. Smoking was also one of three risk factors included in the Batty et al. analysis. We have now included the following text in the manuscript to justify this analysis: *“We chose smoking to test our hypothesis that similar estimates would be derived from both underlying and contributory conditions as smoking is an established risk factor for mortality and it has been used for a similar purpose previously (Batty et al. 2019).”*

 As suggested, we have also now included the estimates for all-cause mortality in Figure 3 and described these results more fully in the text describing that graph.

 Batty GD, Gale CR, Kivimäki M, Bell S. Assessment of Relative Utility of Underlying vs Contributory Causes of Death. JAMA Netw Open. 2019 Jul 3;2(7):e198024. doi: 10.1001/jamanetworkopen.2019.8024. PMID: 31365105; PMCID: PMC6669894.

**Comment 7.** In the results, you mention that "mortality rates were higher among less educated participants, manual occupation social class groups, and those with lower average annual household incomes." I can see in Table 3 that (for example) 53% of deaths were among people with only primary education, while 32% of the baseline sample had only primary education. This does suggest higher mortality rates in this group, but does not explicitly show the rates or the association between education and mortality. I'd suggest either omitting this from the results, or adding specific results that support this association.

**Response 7.** An important purpose of this paper is to provide an overview of the linked mortality data available in TILDA. Indeed, an important deliverable of the mortality project discussed above is the development of a data infrastructure of linked mortality / survey data. We hope that this manuscript will be an important reference for researchers using this new data resource. 

 With this in mind, our intention in including the information in Table 3 was to provide a brief description of decedents within the TILDA sample. We did not intend to suggest associations as such. Indeed, as also described above, explicitly and rigorously testing these associations is a central aim of the project and a number of manuscripts are currently in development that do just that.

 In an effort to make this clearer to readers we have now included the following text: *"For reference, the distribution of important socio-demographic characteristics of the full TILDA sample and those who have died over the course of the study are presented in Table 3."*

**Comment 8.** I like the age-specific comparison to the general population provided in Figure 1. The results say that "Overall, mortality rates among younger TILDA participants aligned closely with those observed in the population. We did however observe some important differences with higher mortality rates observed among older decedents in our sample compared to the wider population."

 However, in the figure, mortality rates look lower for the TILDA participants at both younger and older ages. It may help to (a) plot these charts with a log y-axis, and (b) use a model to plot a smooth curve with confidence limits that can be more easily compared to the general population. It looks like a simple exponential model would work, (c) report the age-standardised mortality rate for both the cohort and the general population.

 Also note that the mortality rate is not among decedents but among the population/participants.

**Response 8.** Our understanding is that the y-axis hazard rates are in effect standardised as described in the text *“* *The mortality rate on the y-axis was based on the hazard function which was calculated as the number of deaths at age x / the number of persons surviving to exact age x out of the original 100,000 aged 0.”*

 That said, we did try to find an alternative means of presenting this comparison as suggested by you. Unfortunately we were unable to create an informative and easily interpreted solution. One difficulty is the small number of deaths observed within years, or indeed age bands. For example, for suggestion b, this leads to massive CIs among older ages in particular.

 Also, the approach we have taken is similar to that of Weir (2016) when validating mortality data for the TILDA sister study, the Health and Retirement Study. Our representation therefore aids comparability of the two studies. We do however appreciate these suggestions and hope to have greater success in our efforts to incorporate them when we repeat this exercise in 2021.

 We have replaced ‘older decedents’ with ‘older ages’ in the offending sentence.

**Comment 9.** In the limitations, you note that "There is necessarily a time lag whereby, unbeknownst to us, participants may have died since the last round of data collection. This is inevitable as we do not have an automated linkage system with the GRO. The practical effect of this is that we have likely underestimated the rates of mortality for the most recent period." It may be possible to address this by ending follow-up at an earlier date, e.g. 6 months before the final linkage date, to increase the likelihood that your study includes all deaths for the follow-up period.

**Response 9. **This is an interesting suggestion. Thank you. TILDA intends to collect its 6 ^th^ wave of data in 2021 and during that time we will repeat this data linkage exercise. We know that there have been a quite a number of deaths since we carried out this exercise and given the large numerator (count of deaths) this will result in, we will consider, as you suggest, trimming our survival time.

**Response to Reviewer 2 comments – Peter Harteloh**

**Comment 1.** Linkage studies are important for enhancing the analytical power of cause-of-death registrations. They provide insight in associations between causes of death and their determinants. Linkage studies improve the utility of cause-of-death registrations for health policy or research. The study of Ward *et al. *is a fine example of such a linkage study. It is clear and well written. It shows associations between social economic status and causes of death both from a traditional approach by selecting one underlying cause of death per deceased and by a multiple cause coding approach. I would surely recommend its indexing, but ask for some minor revisions and answers to some questions.

**Response 1.** We wish to thank Dr Harteloh for his positive review of our manuscript. This is the first time that this data linkage exercise has been conducted in the Republic of Ireland and as such we hope that it will be a valuable resource for researchers who wish to better understand the antecedents of mortality among older adults.

 As also discussed in response to Reviewer 1, this data linkage exercise was the first step in a wider programme of research being conducted within TILDA. This research is funded by the Health Research Board (ILP-PHR-2017-022)

 The project is titled “Do we die as we live? Age, socioeconomic status, healthcare utilisation and pathways to death in Ireland”.

**Comment 2.** Abstract: “Death records were obtained for 779 (90.3% of all confirmed deaths at that time) and linked to individual level survey data from The Irish Longitudinal Study on Ageing (TILDA).” Typo: Close brackets after 90.3% instead of after “time”.

**Response 2. **This has been corrected.

**Comment 3**. Methods. Coding of cause of death: “In our case, Iris successfully coded 18% of the 1,605 diagnostic expressions and assigned an underlying cause to 5.3% of the cases.” Usually about 60-70% of the records are coded automatically: see Harteloh, 2018 . Can the authors explain this poor performance? If the performance of Iris is really that bad, I would not recommend using the software. I would consider the records coded manually. Could the authors say something about the instructions for manual coding i.e. processing the records not being coded automatically by Iris. Are all medical expressions on the death certificate coded and do the coders use volume 2 of the ICD-10? Are there any instructions deviating from volume 2 of the ICD-10 used? (as local certifying practice sometimes requires).

 Also, if a record was rejected by Iris and then handled manually by coding all the expressions on a death certificate, Iris can select the underlying cause of death automatically in most of the cases (about 95%). I wonder why this function of Iris has not been used by the authors? In short, I would like to have some more information about the use of Iris in the coding process in order to understand the multiple cause coding approach of the authors.

**Response 3.** The poor performance of Iris in assigning an ICD-10 code to the conditions mentioned in the individual death records was largely due to the fact that the death records had not been cleaned prior to our receiving them. As these records were provided as strings, their quality / consistency was variable. As this was the first time we had used the Iris software, the generic data dictionary included with the software, failed to identify conditions with different spellings, random spaces, and other typographical errors.

 One recurring example which we believe exemplifies this was the case of “ischemic” / “ischaemic”. The of-the-shelf dictionary in the software correctly identified the former but not the latter, which was in fact the more common spelling in the death certificates. As part of our data processing we appended the in-built data dictionary with common variations of spellings and descriptors we encountered and as a result, Iris performed this task increasingly well as we progressed. We plan to conduct data matching again in 2021 and are confident that we will have a higher success rate in our next attempt to assign ICD-10 codes automatically to individual death records.

 We confirm that we coded the string expressions on the death records according to volume 2 of the ICD-10 with no local deviations.

 Once ICD-10 codes were inputted (either automatically or manually), Iris performed excellently when selecting an underlying cause of death using the decision tables described in the manuscript. Indeed, this is the function that attracted us to using software for this purpose as it removed the possibility of subjective, or coder variation in the assignation of underlying cause.

**Comment 4.** Methods. Data linkage. Can the authors say something about the ethics of linking survey data with cause of death registrations? They seem to suggest (“We grouped underlying causes of death to ICD-10 chapters in order to adhere to TILDA data protection policies regarding minimum cell sizes for reporting purposes”) some ethical restrictions.

 I wonder if the participant of the survey study gave permission for linkage to other data sources such as a cause of death registration.

**Response 4. **TILDA has full ethical approval in place for all data collection waves and further gains informed consent from all participants prior to data collection. Ethical approval is approved by the Faculty of Health Sciences Research Ethics Committee, Trinity College Dublin. Participants are informed through the Participant Information Leaflet that their data is shared in a confidential manner as part of the TILDA study.

 The TILDA Privacy Policy gives more detailed information about data linkage with the GRO. It is important to note also that GDPR and the Irish Health Research Regulations do not apply to the personal data of deceased individuals. For the situation where a participant may be lost to follow up and their status unknown, TILDA have been granted a consent declaration by the Health Research Consent Declaration Committee (HRCDC) to process their data for GRO Linkage. A HRCDC declaration is granted in a case where the public interest of doing the research significantly outweighs the need for explicit consent.

 A data transfer agreement is signed between TCD and GRO which commits to protecting the confidentiality of data. Physical and technical safeguards are also in place.

**Comment 5. **Methods. A definition (explanation) of “contributory cause of death” is missing. It is commonly defined as a cause of death, not being selected as underlying cause of death (and mentioned in part 2 of the death certificate). However, the authors seem to use it for causes of death being mentioned on a death certificate. Otherwise, I cannot understand so many malignancies not being underlying cause of death (see table 4).  So please explain the use of this concept (or replace it by “being mentioned”, regardless of being underlying cause of death)

**Response 5.** Our use of the term ‘contributory’ was informed by a study by Batty et al.  who use the term to refer to “Other diseases or injuries that contributed to the death but were not directly implicated” (p.2).

 We have now explained our use of the term ‘contributory’ and provided a reference to the Batty et al. paper. *“A contributory cause of death is a condition that contributed to the death but were not directly implicated and are recorded in part two of death certificates. While this information has been rarely used in epidemiological research, recent evidence suggest it may have some methodological utility (Batty et al. 2019). For present purposes, contributory causes include diseases and conditions listed anywhere on the death certificate.”*

 Batty GD, Gale CR, Kivimäki M, Bell S. Assessment of Relative Utility of Underlying vs Contributory Causes of Death. JAMA Netw Open. 2019 Jul 3;2(7):e198024. doi: 10.1001/jamanetworkopen.2019.8024. PMID: 31365105; PMCID: PMC6669894.

**Comment 6.** Methods. Why did the authors (specifically) focus on the relationship between smoking and causes of death? What about other SES determinants? In order to avoid fishing expeditions, the selection of determinants to be studied should be clearly motivated.

**Response 6. **This valid point was also raised by another reviewer. In response, this particular analysis was informed by similar work carried out using UK Biobank data by Batty et al. The aim of this research, and our aim also, was to assess the utility of cause of death data extracted from the underlying cause field versus any location on the death certificate.

 Our choice of smoking as a risk factor was, as you identify, because it is so well established. Smoking was also one of three risk factors included in the Batty et al. analysis. We have now included the following text in the manuscript to justify this analysis: *“We chose smoking to test our hypothesis that similar estimates would be derived from both underlying and contributory conditions as smoking is an established risk factor for mortality and it has been used for a similar purpose previously (Batty et al. 2019).” *Batty GD, Gale CR, Kivimäki M, Bell S. Assessment of Relative Utility of Underlying vs Contributory Causes of Death. JAMA Netw Open. 2019 Jul 3;2(7):e198024. doi: 10.1001/jamanetworkopen.2019.8024. PMID: 31365105; PMCID: PMC6669894.

**Comment 7.** Results. “while diseases of the circulatory system and diseases of the respiratory system were mentioned in 52.6% and 34.4% respectively”. Did the authors count records mentioning at least one cause of death of the group under consideration?

**Response 7. **We hope we have interpreted this question correctly, but we confirm that the figures refer to the proportion of death certificates that included any cause from the ICD-10 chapter of diseases of the circulatory system as a contributory cause of death (52.6 %) and any cause from the ICD-10 chapter of diseases of the circulatory system (34.4%).

**Comment 8.** Results. Table 4. I think mentioned (of a death record) instead of contributory cause of death is meant here. Also in the column counting contributory causes of death: is this a count of records mentioning at least one malignancy etc… Otherwise, the numbers seem very low to me.

**Response 8.** Yes. This is a count of records that included at least one malignancy per record.

 We hope that the additional text we have included in response to your comment 5 in defining our use of ‘contributory’ has made this clearer to readers.

**Comment 9.** Results. Figure 3. Very interesting approach. Could the authors explain the fact that smoking is not a statistically significant determinant of cancer death? I assume lung cancer is the most prevalent cancer as cause of death.

**Response 9.** Lung cancer was indeed the most common type accounting for 19% of cancers. We note that the association between smoking and cancer death is positive, but non-significant due to wide 95% confidence bands. We also note that our smoking variable identifies ‘ever’ as well as ‘current’ smokers, so some of the smokers may have quit some time ago.

**Comment 10.** Results. “In each instance, we observed similar estimates whether we assigned death due to an underlying or contributory cause.” Not clear. Please explain or show these estimates.

**Response 10.** These estimates (HRs with 95% CIs ) are presented in Figure 3. In responses to another reviewers suggestion, we have now also included the estimates for all-cause mortality. We also now more fully describe the results presented in this figure. We hope that this fuller description also provides clearer support for our contention that choice of contributory or underlying cause may not make much difference to these estimates. This final point is more fully discussed in response to comment 11 below and comment 6 from Reviewer 1.

**Comment 11.** Results. “We observed similar estimates whether we assigned death due to an underlying or contributory cause, which suggests the use of either contributory or underlying cause may not greatly impact on estimates of the association between risk factors and mortality. “ A bit far fetched for such an important conclusion when the estimates are not shown.

 In addition, could the negative result be explained by the grouping of causes of death? I would like to see the result of associations between risk factors and major causes of death such as dementia, lung cancer or cerebrovascular accidents if the privacy rules are not violated.

**Response 11.** As in our response to the previous comment, these estimates are presented in Figure 3 and the text describing these results has been extended.

 Our contention that it appears that underlying and contributory cause of death may have similar utility for studies examining mortality risk factors is supported by the work discussed above by Batty et al. (2019) and a smaller scale study by Crews et al. (1991). We have now referenced both of these studies in support of the contention we made here. 

 We are also going to repeat the data linkage exercise in 2021 when TILDA will conduct its 6 ^th^ wave of data collection. The increased number of deaths will provide us with an appropriately large sample size to examine the association of major risk factors and specific causes of death. Initial results from this work are anticipated in late 2021.

**Comment 12.** Discussion. “For example, Iris failed to automatically code cases of “ischaemic heart disease” as it searched for “ischemic”. This example is not clear to me. When you put “ischaemic heart disease” in your dictionary Iris will be able to code the expression automatically. Please explain.

**Response 12.** We have again checked this and can confirm that the Iris data dictionary does not identify “ischaemic heart disease”, only “ischemic heart disease”. The reason we chose to refer to this example was because it occurred so often.

 As part of our data processing we appended the in-built data dictionary with common variations of spellings and descriptors we encountered and as a result, Iris performed this task increasingly well as we progressed. We plan to conduct data matching again in 2021 and are confident that we will have a higher success rate in our next attempt to assign ICD-10 codes automatically to individual death records.

**Comment 13.** Conclusion. “This is the first time that death registration data has been linked to survey data in the Republic of Ireland. This work therefore provides an important data infrastructure for research on mortality in Ireland.“ I agree! This is a very important aspect of this study. It deserves to be indexed.

**Response 13.** Thank you. We are glad that you agree with the importance of this exercise. As described above, we hope that project that this work stems from will make an important contribution to research on mortality in Ireland. We also hope that this particular data linkage demonstrates the great potential of combining rich individual level survey data with administrative data sources. Unfortunately, to date Ireland somewhat lags behind other jurisdictions who have well developed data linkage infrastructures.

**Comment 14.** Outcome of my review: approved. Some minor issues to be addressed. Most important: clear up the use of the term “contributory cause of death”. Finally, I would like to compliment the authors on their research and encourage further analysis.

**Response 14.** Again, we wish to thank Dr Harteloh for his constructive feedback. We believe that the revisions have greatly improved the manuscript and provided clarification as to the meaning of contributory cause in this context. As discussed above, this is the first of many publications from this work. If interested, we have recently published another methodological paper using this data which compares the utility of cause of death data from official records and reports from end-of-life interviews.  Ward, M, May, P, Normand, C, Kenny, RA, and Nolan, A. Comparing Underlying and Contributory Cause of Death in Registry Data With End-of-Life Proxy Interviews: Findings From The Irish Longitudinal Study on Ageing (TILDA). Journal of Applied Gerontology. [In Press]. https://doi.org/10.1177/0733464820935295

**Response to Reviewer 3 comments – Dr Zubair Kabir**

**Comment 1. **This is an important piece of linkage study that is relevant to the Irish context when such data linkages are available elsewhere. It is also important to note that linkage studies are methodologically challenging in Ireland because of the lack of a unique identifier. The CSO did make attempts earlier to undertake such linkage research but was insufficient and was both labour and resource intensive. The current study builds on earlier linkage studies undertaken both by CSO and GRO in 2013 and 2018, respectively.

**Response 1.** Thank you Dr Kabir for taking the time to review our manuscript and for your helpful observations. As you rightly say, this type of exercise is challenging within the Irish data infrastructure and we do hope that our efforts contribute to improving this situation.

**Comment 2.** My main concern is the lack of explicit description of the linkage methodology in the current paper, which will not be very helpful for a researcher towards reproducibility. There are currently no standardized quality appraisal tools available to assess quality and bias of any linkage studies. However, it is essential that a linkage study must meet the following characteristics:

 Completeness of source databases; Accuracy of data sources; Linkage methodology and technology;

 Ethical and data security considerations.

 In the context of the current study - the first two criteria are broadly met. However, my main concern is with the linkage methodology and technology. My understanding is that the TILDA researchers were not primarily involved in the linkage methodology given that matching of records were undertaken separately by CSO in 2013 and by GRO in 2018. The TILDA team had a role to get an approval and forward their data to these two data sources team who in fact undertook the matching process - the details of which are not available to us.

 It also appears that the technology (software) used is IRIS, which is a broadly validated accepted tool for coding purposes employed by EUROSTAT and CSO in the past. However, this software also had limitations in capturing and coding all the diagnostic expressions - only 18% and 5% of all the cases. The rest of the matching was done manually - by whom and how is unclear. This is a crucial step for which sufficient information and clarity is lacking. Second, the matching was not 100% accurate - around 10% of records were unmatched - and further analyses of these unmatched records are

 essential to rule out systematic bias - measurement error, and such sensitivity analyses (false positives and false negatives) have not been provided. Third, the matching variables employed were only three - name, address, and age (and marital status for some, but not sure for how many?). Names, especially for females can change once married; addresses are not always permanent - and age is also variable.

 Therefore, further details on how these methodological limitations during the process of matching were handled are unclear. There is also limited information on ethical and data security considerations for this linkage study when personal data have been used, especially from a GDPR perspective.

**Response 2. **We have done our best to describe as fully as possible the steps we took to achieve this data linkage. We hope that our responses to yours’ and other reviewers suggestions have further improved this.

 Naturally, many of our decisions and subsequent actions are specific to the data environment in which the work was conducted. By this, we mean that we were confined to the data that was available to use in TILDA, for example, the individual identifiers and so on. As such, it may well not be possible to replicate our procedures with other studies in Ireland. However, we feel strongly that we have been fully transparent and as specific as possible in our description of the steps we have taken to link the individual-level survey data available in TILDA to official death records. Indeed, given the richness of the data available to us in TILDA, we have many advantages not necessarily available to other studies. 

 As you correctly state, there are no standardised quality assurance tools available to use to assess the validity of our data linkage procedures and it was partly due to the absence of such a tool that we felt compelled to describe our methods as fully as possible and importantly to make this manuscript freely available to all.

 Also importantly, our intention with this manuscript was not to suggest a one-size fits all method but rather to describe a new data infrastructure within TILDA that researchers interested in studying mortality in Ireland might avail of. How a similar task might be approached using a different study sample will be study dependent. That said, we do believe that our use of the Iris software tool for coding and identifying underlying cause of death is one way in which our work might be replicated and could help ensure standardisation in at least this aspect of the linkage across studies.

Completeness of source databases

 As TILDA is prospective cohort study we are confident of the accuracy of the participant contact information and status as participants are contacted regularly and the status of non-responders is followed up via the participants or their proxies. The contact database is regularly updated so that participants can be contacted for future rounds of data collection.

 The GRO is the official register of all deaths in Ireland and provides information on deaths to the CSO for use in official statistics. As such, we are confident that it is a reliable and comprehensive source of data on deaths in Ireland.

Ethical and data security considerations

 TILDA has full ethical approval in place for all data collection waves and further gains informed consent from all participants prior to data collection. Ethical approval is approved by the Faculty of Health Sciences REC, Trinity College Dublin. Participants are informed through the Participant Information Leaflet that their data is shared in a confidential manner as part of the TILDA study.

 It is important to note also that GDPR and the Irish Health Research Regulations do not apply to the personal data of deceased individuals. For the situation where a participant may be lost to follow up and their status unknown, TILDA have been granted a consent declaration by the Health Research Consent Declaration Committee to process their data for GRO Linkage. A HRCDC declaration is granted in a case where the public interest of doing the research significantly outweighs the need for explicit consent.

 A data transfer agreement is signed between TCD and GRO which commits to protecting the confidentiality of data. Physical and technical safeguards are also in place.

Linkage methodology and technology

 Our stating  that data matching was conducted by the CSO in 2013 was in error and has now been removed from the manuscript. The only time data matching took place was in 2018 with the GRO.

 The TILDA data team did undertake the data matching through the GRO search room facility. Once the TILDA team member identified the decedent within these records, the GRO then provided the detailed death certificate information for this person.

 We have provided further clarification to these points in response to earlier comments. We have also appended our description of these measures within the manuscript and hope that they adequately address each of the points raised here.

 You may also be interested to know that the CSO have repeated their 2013 data linkage using 2016 census data. You will find the results here: CSO: Mortality Differentials in Ireland. An Analysis Based on the Census Characteristics of Persons Who Died in the Twelve Month Period after Census Day 24 April 2016. 2019. Dublin. Source: https://www.cso.ie/en/releasesandpublications/in/mdi/mortalitydifferentialsinireland2016-2017/ [Accessed: October 2020].

**Comment 3. **Furthermore, the coding practices of causes of death are crucial for any linkage studies. The authors have undertaken a separate analysis of exploring contributory versus underlying causes of deaths for the participants, and I believe that this piece of research is the sole contribution of the TILDA team to this paper.

 However, this could have been explained further and there is lack of clarity on how the unclassified causes of deaths within each of the three main types of causes of deaths (cancer, cardiovascular and respiratory) were handled. The CSO website clearly indicates ‘unclassified’ causes of cancer deaths and likewise for other conditions - and the Global Burden of Disease (GBD) Study team call these as ‘garbage’ codes. The GBD studies on causes of death have shown that there is a good proportion of ‘garbage’ codes for any death registry, and they have also developed a statistical technique on how to ‘redistribute’ these garbage codes. No such information is available to us in the current study.

 In short, I approve the study but has methodological limitations and caveats which could have been

 addressed.****

**Response 3. **We hope we have clarified that the full data linkage exercise was conducted by the TILDA team. In practice the GROs sole involvement was to provide the team with the death certificate information of decedents identified among TILDA participants.

 In light of these, we believe we have made three contributions here. (1) We performed the data linkage, (2) provided an overview of a new data infrastructure and, (3) provided an assessment of the utility of contributory versus underlying cause in estimating the association between risk factors and mortality risk.

 As also detailed in response to Reviewers 1 and 2 above, in this amended version of the manuscript we have better described our use of the term ‘contributory’ as: *“A contributory cause of death is a condition that contributed to the death but were not directly implicated and are recorded in part two of death certificates. While this information has been rarely used in epidemiological research, recent evidence suggest it may have some methodological utility (Batty et al. 2019). For present purposes, contributory causes include diseases and conditions listed anywhere on the death certificate.”*

 Batty GD, Gale CR, Kivimäki M, Bell S. Assessment of Relative Utility of Underlying vs Contributory Causes of Death. JAMA Netw Open. 2019 Jul 3;2(7):e198024. doi: 10.1001/jamanetworkopen.2019.8024. PMID: 31365105; PMCID: PMC6669894.

 Also to re-state an earlier response, this particular analysis was informed by similar work carried out using UK Biobank data by Batty et al. The aim of this research, and our aim also, was to assess the utility of cause of death data extracted from the underlying cause field versus any location on the death certificate. The estimates do also confirm a stronger association between smoking and respiratory causes of death compared to all-cause mortality which is re-assuring but was not our main aim in this analysis.

 Our choice of smoking as a risk factor was, as you identify, because it is so well established. Smoking was also one of three risk factors included in the Batty et al. analysis. We have now included the following text in the manuscript to justify this analysis: *“We chose smoking to test our hypothesis that similar estimates would be derived from both underlying and contributory conditions as smoking is an established risk factor for mortality and it has been used for a similar purpose previously (Batty et al. 2019).”* Again, we sincerely thank Dr Kabir for his insightful comments and appreciate his sharing his vast experience in this area with us.

# Peer review: Reviewer response for version 1

**DOI:** 10.21956/hrbopenres.14183.r27634

**Published:** 2020-07-22

**Authors:** Peter Harteloh

**Affiliations:** 1 Statistics Netherlands (CBS), The Hague, The Netherlands

Linkage studies are important for enhancing the analytical power of cause-of-death registrations. They provide insight in associations between causes of death and their determinants. Linkage studies improve the utility of cause-of-death registrations for health policy or research. The study of Ward *et al.* is a fine example of such a linkage study. It is clear and well written. It shows associations between social economic status and causes of death both from a traditional approach by selecting one underlying cause of death per deceased and by a multiple cause coding approach. I would surely recommend its indexing, but ask for some minor revisions and answers to some questions.

 Abstract: “Death records were obtained for 779 (90.3% of all confirmed deaths at that time) and linked to individual level survey data from The Irish Longitudinal Study on Ageing (TILDA).” Typo: Close brackets after 90.3% in stead of after “time”.

 Methods. Coding of cause of death: “In our case, Iris successfully coded 18% of the 1,605 diagnostic expressions and assigned an underlying cause to 5.3% of the cases.” Usually about 60-70% of the records are coded automatically: see Harteloh, 2018 ^1^. Can the authors explain this poor performance? If the performance of Iris is really that bad, I would not recommend using the software. I would consider the records coded manually. Could the authors say something about the instructions for manual coding i.e. processing the records not being coded automatically by Iris. Are all medical expressions on the death certificate coded and do the coders use volume 2 of the ICD-10? Are there any instructions deviating from volume 2 of the ICD-10 used? (as local certifying practice sometimes requires).

 Also, if a record was rejected by Iris and then handled manually by coding all the expressions on a death certificate, Iris can select the underlying cause of death automatically in most of the cases (about 95%). I wonder why this function of Iris has not been used by the authors?

 In short, I would like to have some more information about the use of Iris in the coding process in order to understand the multiple cause coding approach of the authors.

 Methods. Data linkage. Can the authors say something about the ethics of linking survey data with cause of death registrations? They seem to suggest (“We grouped underlying causes of death to ICD-10 chapters in order to adhere to TILDA data protection policies regarding minimum cell sizes for reporting purposes”) some ethical restrictions. I wonder if the participant of the survey study gave permission for linkage to other data sources such as a cause of death registration.

 Methods. A definition (explanation) of “contributory cause of death” is missing. It is commonly defined as a cause of death, not being selected as underlying cause of death (and mentioned in part 2 of the death certificate). However, the authors seem to use it for causes of death being mentioned on a death certificate. Otherwise, I cannot understand so many malignancies not being underlying cause of death (see table 4). So please explain the use of this concept (or replace it by “being mentioned”, regardless of being underlying cause of death).

 Methods. Why did the authors (specifically) focus on the relationship between smoking and causes of death? What about other SES determinants? In order to avoid fishing expeditions, the selection of determinants to be studied should be clearly motivated.

 Results. “while diseases of the circulatory system and diseases of the respiratory system were mentioned in 52.6% and 34.4% respectively”. Did the authors count records mentioning at least one cause of death of the group under consideration?

 Results. Table 4. I think mentioned (of a death record) instead of contributory cause of death is meant here. Also in the column counting contributory causes of death: is this a count of records mentioning at least one malignancy etc… Otherwise, the numbers seem very low to me.

 Results. Figure 3. Very interesting approach. Could the authors explain the fact that smoking is not a statistically significant determinant of cancer death? I assume lung cancer is the most prevalent cancer as cause of death.

 Results. “In each instance, we observed similar estimates whether we assigned death due to an underlying or contributory cause.” Not clear. Please explain or show these estimates.

 Results. “We observed similar estimates whether we assigned death due to an underlying or contributory cause, which suggests the use of either contributory or underlying cause may not greatly impact on estimates of the association between risk factors and mortality. “ A bit far fetched for such an important conclusion when the estimates are not shown. In addition, could the negative result be explained by the grouping of causes of death? I would like to see the result of associations between risk factors and major causes of death such as dementia, lung cancer or cerebrovascular accidents if the privacy rules are not violated.

 Discussion. “For example, Iris failed to automatically code cases of “ischaemic heart disease” as it searched for “ischemic”. This example is not clear to me. When you put “ischaemic heart disease” in your dictionary Iris will be able to code the expression automatically. Please explain.

 Conclusion. “This is the first time that death registration data has been linked to survey data in the Republic of Ireland. This work therefore provides an important data infrastructure for research on mortality in Ireland.“ I agree! This is a very important aspect of this study. It deserves to be indexed.

 Outcome of my review: approved. Some minor issues to be addressed. Most important: clear up the use of the term “contributory cause of death”. Finally, I would like to compliment the authors on their research and encourage further analysis.

Is the rationale for developing the new method (or application) clearly explained?

Yes

Is the description of the method technically sound?

Yes

Are the conclusions about the method and its performance adequately supported by the findings presented in the article?

Yes

If any results are presented, are all the source data underlying the results available to ensure full reproducibility?

No source data required

Are sufficient details provided to allow replication of the method development and its use by others?

Yes

Reviewer Expertise:

NA

I confirm that I have read this submission and believe that I have an appropriate level of expertise to confirm that it is of an acceptable scientific standard.

## References

[1] : The implementation of an automated coding system for cause-of-death statistics. *Inform Health Soc Care*.2020;45(1) : 10.1080/17538157.2018.1496092 1-14 10.1080/17538157.2018.1496092 30125131

# Peer review: Reviewer response for version 1

**DOI:** 10.21956/hrbopenres.14183.r27635

**Published:** 2020-07-13

**Authors:** Dan Lewer

**Affiliations:** 1 Department of Epidemiology and Public Health, University College London, London, UK

Thank you for inviting me to review this article. It provides a clear summary of a linkage exercise conducted between a community health survey of older people and national mortality data in Ireland.

 The data is a valuable resource and researchers will find this technical article useful.

 To my knowledge this type of data is not commonplace (as per first line of introduction), which strengthens the international importance of this data.

 I think a central use of this data is analyses of the association between longitudinal information on exposures and mortality (e.g. what is the effect of weight loss, quitting smoking, or cognitive decline?). This is not discussed in the article, and I think it might be worth mentioning this as a potential use of the dataset. In general, I would find it useful to know some of the key research questions that the authors think the dataset might address (though of course it's not possible to anticipate all the different research uses).

 A few questions/comments:

1. What is a confirmed death? If not from the linked mortality records, how do you find out that a participant has died (i.e. how do you know that 863 participants died?). Apologies if I missed an explanation of this in the text.
2. Is it worth adding some information on the associations with successful linkage? (i.e. were certain types of participant less likely to be linked?)
3. For participants who are linked, what is the probability of correct linkage? Did the linkage process use an existing method, and is there any validation that the linkages are correct?
4. I like the analysis of smoking. It might be worth adding a brief justification for this analysis to the introduction (e.g. that the relationship between smoking and different causes of death is well-researched in other sources, so it acts as a kind of validation - you would expect a stronger association between smoking and respiratory causes of death than between smoking and all-cause mortality; or because it allows you to evaluate the difference between the derived 'underlying cause' of deaths and contributing causes?). Would it be possible to add the association between ever-smoking and all-cause mortality to figure 3 for comparison?
5. In the results, you mention that "mortality rates were higher among less educated participants, manual occupation social class groups, and those with lower average annual household incomes." I can see in Table 3 that (for example) 53% of deaths were among people with only primary education, while 32% of the baseline sample had only primary education. This does suggest higher mortality rates in this group, but does not explicitly show the rates or the association between education and mortality. I'd suggest either omitting this from the results, or adding specific results that support this association.
6. I like the age-specific comparison to the general population provided in Figure 1. The results say that "Overall, mortality rates among younger TILDA participants aligned closely with those observed in the population. We did however observe some important differences with higher mortality rates observed among older decedents in our sample compared to the wider population." However, in the figure, mortality rates look lower for the TILDA participants at both younger and older ages. It may help to (a) plot these charts with a log y-axis, and (b) use a model to plot a smooth curve with confidence limits that can be more easily compared to the general population. It looks like a simple exponential model would work, (c) report the age-standardised mortality rate for both the cohort and the general population. Also note that the mortality rate is not among decedents but among the population/participants.
7. In the limitations, you note that "There is necessarily a time lag whereby, unbeknownst to us, participants may have died since the last round of data collection. This is inevitable as we do not have an automated linkage system with the GRO. The practical effect of this is that we have likely underestimated the rates of mortality for the most recent period." It may be possible to address this by ending follow-up at an earlier date, e.g. 6 months before the final linkage date, to increase the likelihood that your study includes all deaths for the follow-up period.

Is the rationale for developing the new method (or application) clearly explained?

Yes

Is the description of the method technically sound?

Yes

Are the conclusions about the method and its performance adequately supported by the findings presented in the article?

Yes

If any results are presented, are all the source data underlying the results available to ensure full reproducibility?

Yes

Are sufficient details provided to allow replication of the method development and its use by others?

Yes

Reviewer Expertise:

Research using electronic health records; public health; health and social exclusion; health inequalities.

I confirm that I have read this submission and believe that I have an appropriate level of expertise to confirm that it is of an acceptable scientific standard.
